# Feasibility and Complications between Phacoemulsification and Manual Small Incision Surgery in Subluxated Cataract

**DOI:** 10.1155/2012/205139

**Published:** 2012-03-05

**Authors:** Ruchi Goel, Saurabh Kamal, Sushil Kumar, Jugal Kishore, K. P. S. Malik, Sonam Angmo Bodh, Smriti Bansal, Madhu Singh

**Affiliations:** ^1^Guru Nanak Eye Centre, Maulana Azad Medical College, New Delhi 110002, India; ^2^Department of Community Medicine, Maulana Azad Medical College, New Delhi 110002, India; ^3^Department of Ophthalmology, Subharti Medical College, Meerut 250002, India

## Abstract

*Purpose*. To compare the feasibility of cataract surgery with implantation of endocapsular supporting devices and intraocular lens (IOL) in subluxated cataract in phacoemulsification and manual small incision cataract surgery (MSICS). *Design*. Prospective randomized intervention case series consisting of 60 eyes with visually significant subluxated cataract. *Method*. The patients were randomly distributed between the two groups equally. The main outcome measure was implantation of in-the-bag IOL, requirement of additional procedure and complications, if any. *Results*. Capsular bag retention in subluxated lenses is possible in 90% cases in phacoemulsification versus 76.67% cases in MSICS (*P* = 0.16). Both groups, achieved similar best corrected visual acuity (*P* = 0.73), although additional procedures, intraoperative, and postoperative complications were more common in MSICS. *Conclusions*. Achieving intact capsulorhexis and nuclear rotation in MSICS may be difficult in cases with large nucleus size and severe subluxation, but subluxated cataracts can be effectively managed by both phacoemuslification and MSICS.

## 1. Introduction

Subluxated lenses present a serious challenge to every cataract surgeon. The causes of subluxation of the lens include trauma, Marfan's syndrome, Weill-Marchesani syndrome, homocystinuria, idiopathic, and hereditary cases. With the development of newer techniques and devices, complications in these cases have been reduced [1, 2]. These devices include capsular tension ring (CTR), modified CTR with single or double fixation point [1–3], capsular tension segment (CTS) [4], and recently introduced capsular anchor device [5]. Additionally, the use of iris hooks [6] has further improved the stabilization of capsular bag during the cataract surgery. 

Manual small incision cataract surgery (MSICS) and phacoemulsification are the two widely practiced surgical procedures for cataract extraction.

The various techniques of MSICS include wire loop, phaco-sandwich, and phacosection technique [7]. The novel innovation of anterior chamber maintainer (ACM) by Blumenthal and Moisseiev [8] permits a high-pressure and high-flow system, providing a physiological environment throughout the surgery requiring minimal intraocular instrumentation. The procedure, with an initial learning curve, is highly effective, applicable to all grades of cataracts, has minimum intraocular instrumentation resulting in an early rehabilitation of the patient [7]. The MSICS has become popular in India and has been found to be effective and economical [9, 10] requiring less capital investment although phacoemulsification gives better unaided visual acuity [11]. Therefore, this study was carried out with the aim of comparing the feasibility and complications of cataract surgery with endocapsular supporting devices and intraocular lens implantation in subluxated cataract between phacoemulsification and MSICS.

## 2. Materials and Methods

This was a prospective, interventional, consecutive case series performed at the Guru Nanak Eye Centre, New Delhi. Sixty eyes with subluxated cataract, who presented between January 2007 and March 2011, were enrolled consecutively and randomly distributed in two equal groups with thirty patients each. In group A, phacoemulsification and in group B, manual small incision cataract surgery was done with implantation of posterior chamber intraocular lens (IOL) and use of endocapsular supporting device as required. The duration of follow-up period was three months. The ethical committee approved this study. The experiment was conducted with the understanding and the consent of the human subject.

### 2.1. Preoperative Assessment

All patients with subluxated crystalline lens with visually significant cataract were evaluated for cataract surgery. The assessment included evaluation of visual acuity, refractive error, and intraocular pressure (IOP). The biomicroscopy was performed for grading of nuclear sclerosis, degree of subluxation with zonular loss or any weakness, vitreous in anterior chamber, and injury to any other ocular structure. The imaging included ultrasound biomicroscopy for anterior chamber angle and zonular status and B-scan for posterior segment evaluation. The exclusion criteria included age less than 18 yrs, intraocular pressure more than 21 mm of Hg, scleral thinning, more than 210 degree subluxation, pseudoexfoliation, active uveitis, corneal opacity/scarring, and history of open globe injury and retinal detachment.

The degree of subluxation was divided into three groups (mild <90, moderate 90–180, and severe >180 degree). The different endocapsular supporting devices were used according to the extent of zonulysis based on the decentration/tilt of the lens preoperatively as well as its mobility intraoperatively. A capsular tension ring (CTR) was used for mild degree of zonulysis, a single-point fixation capsule device (Cionni modified CTR/single Cionni) was used for moderate degree, and for cases with severe degree, a two-point fixation capsule device was used. In case of inadvertent capsular injury, the endocapsular supporting device was not used and transscleral suture fixation of PMMA IOL was carried out. In all other cases, foldable, hydrophilic acrylic IOL was used.

A written informed consent of every patient was taken before enrolment into the study. All the surgeries were performed under local anaesthesia using mixture of 3 mL lignocaine 2% and 3 mL bupivacaine 0.5% by a single surgeon (R.G.), competent in both the MSICS and phacoemulsification.

### 2.2. Surgical Technique

Depending on the site of zonulysis, the wound was constructed either superiorly or temporally. In group A, a clear corneal incision of 3.0 mm was made and anterior vitrectomy was done to remove any vitreous in the anterior chamber. Anterior capsulorhexis of adequate size (5–5.5 mm) was carried out using forceps and high-molecular weight viscoelastic device. Cortical cleavage hydrodissection and nuclear rotation was then done. Endocapsular supporting device was inserted depending upon the degree of zonulysis. Disposable nylon hooks to stabilize the capsular bag were placed in patients with >180 degree zonulysis. Phacoaspiration was then performed and posterior chamber IOL implantation was done. In group B, MSICS was performed using 6.0–6.5 mm sclerocorneal tunnel using modified Blumenthal technique. The anterior chamber maintainer (ACM), a hollow steel tube with a 0.9 mm outer diameter and 0.65 mm inner diameter [7], was fixed away from the site of zonulysis. The nucleus was prolapsed out of the bag and then out of the tunnel using assisted delivery, if required. Cortical clean up was carried out, then IOL was implanted and using figure of infinity the section was sutured.

Any additional procedure required in two groups was recorded. The procedures like anterior vitrectomy, pupilloplasty, and iridodialysis repair were labelled as minor and procedures like lensectomy with transscleral suture fixation of IOL (SFIOL) was labelled as major. Similarly any intraoperative complications were recorded.

### 2.3. Postoperative Followup

The patients were followed up for a period of three months, and following parameters were recorded: visual acuity, refractive error, IOL centration/tilt and complications if any were noted. The method of Guyton and coauthors [12] and the formula of Kozaki and coauthors [13] were used to calculate IOL decentration and tilt.

### 2.4. Outcome Measures

The main outcome measures were the feasibility and success of performing the cataract surgery with use of endocapsular supporting devices and IOL implantation in two groups. The occurrence of intraoperative complications and requirement of major additional procedure were compared. Postoperative complications, best corrected visual acuity (BCVA), and IOL decentration/tilt were also compared. The statistical analysis was carried out using Mann Whitney test (for parametric data) and Mantel Haenszel and Fisher exact test (for nonparametric data).

## 3. Results

### 3.1. Preoperative Comparison

The Table 1 shows the comparison of the two groups. The mean age at surgery in group A (Phacoemulsification) was 41.80 ± 12.80 years and group B (MSICS) was 39.86 ± 12.75 years. The majority of the patients in both the group were male, 24 (80%) in group A and 20 (66.7%) in group B.

For the statistical analysis the decimal acuity was used. The mean visual acuity in group A was 0.14 ± 0.10 and in group B was 0.13 ± 0.10, with Kruskal-Wallis *H* value of 0.01 and *P* value of 0.91. The majority of the patients had a moderate degree of subluxation, 18 (60%) in group A and 17 (56.67%) in group B. Between the two groups, the *P* value of mild and moderate type of subluxation was calculated using the Mantel Haenszel test, and for severe subluxation, Fisher exact test was used due to few number of cases. The groups were statistically comparable.

In majority of the cases, the cause of subluxation was trauma (56.7% in group A and 70% in group B) followed by idiopathic (36.7% in group A and 20% in group B). The congenital causes included the Marfans syndrome in two patients in group A and three patients in group B. The groups were statistically comparable.

Table 1 also shows the distribution of the patients according to the grade of nucleus in the two groups. The majority of the patients, 19 (63.33%) in group A and 20 (67.67%) in group B had grade of 2+ or less. The mean value was 2.2 ± 0.80 in group A and 2.13 ± 0.81 in group B (Kruskal-Wallis *H* value of 0.10 and *P* value of 0.74).

### 3.2. Postoperative Comparison

Table 2 shows the postoperative comparison of two groups. In group A, the implantation of the endocapsular supporting device and in the bag IOL was successful in 27 (90.0%) patients, while in group B, it was successful in 23 (76.67%) patients. This difference was statistically insignificant (*χ* ^2^ = 1.89, *P*  value = 0.16).

The comparison of groups for the implantation of an intended endocapsular devices showed that in 25 (83.33%) patients in group A and 20 (66.67%) patients in group B it was successful. This difference was statistically insignificant (*χ* ^2^ = 2.19, *P*  value = 0.14). In group A, 3 cases required transscleral suture fixation of IOL and 2 cases required two-point fixation CTR in place of single fixation device (due to increased dehiscence during chopping). In group B, 7 cases required transscleral suture fixation of IOL and 3 cases required two point fixation CTR in place of single fixation device. One case in group B had increase in dehiscence from 100 to 160 degree intraoperatively and intended single fixation capsule device was implanted.

The comparison of groups in relation to requirement of additional procedure showed that 9 (30%) and 13 (43.33%) cases required it in groups A and B, respectively. Though the number of these procedure were more in group B, but it was not significant (*χ* ^2^ = 1.15, *P*  value = 0.28). Similarly, more intraoperative complications were noted in group B, 36.67% versus 20% but this was insignificant (*χ* ^2^ = 2.02, *P*  value = 0.15).

The mean BCVA achieved in groups A and B was 0.66 ± 0.23 and 0.68 ± 0.28, respectively (*χ*2 = 0.11, *P*  value = 0.73). The mean postoperative spherical and cylindrical error, in group A, was 0.11 ± 0.25 D and 0.58 ± 0.43 D, respectively, and group B was 0.15 ± 0.33 D and 0.95 ± 0.48 D, respectively.

The IOL decentration of >1 mm and tilt of >15 degree was considered significant. The IOL decentration developed in one patient in group B. In this case, dehiscence increased to 210 degree from 160 degree intraoperatively and implantation of two-point fixation CTR was carried out. The IOL tilt was noticed in one patient in group A, where 200 degree of subluxation was present and implantation of two-point fixation device was done.

## 4. Discussion

The success of cataract surgery in subluxated cataract depends upon the ability to implant an endocapsular supporting device and in-the-bag IOL. Historically, surgical removal of the subluxated lens has been undertaken with great caution because of numerous reports of complications and poor visual outcomes. The intracapsular cataract surgery is avoided in such patients due to risk of vitreous loss, retinal detachment, and persistent inflammation as well as anterior chamber IOL-(ACIOL-) related complications [14]. Retention of capsular bag is preferred, unless the lens is dislocated in the posterior vitreous, where pars plana lensectomy is indicated. When the posterior capsule ruptures or there is lack of zonular support, an IOL can be placed in the anterior chamber between the cornea and iris, as in iris-fixated and closed or open-loop ACIOL, or it can be implanted in the posterior chamber within the ciliary sulcus posterior to the iris, as in sutured iris-fixated and scleral fixated posterior chamber IOL. Because of their anatomic advantage, SFIOL provides better visual acuity and binocularity and avoids the complications of ACIOLs, which are seen more with rigid closed loop IOLs than with open-loop and iris-claw IOLs [15].

During cataract surgery in patients with subluxated lens, further damage to compromised zonules is achieved by use of dispersive viscoelastics, bimanual method of capsulorhexis using forceps and cystitome, cortical cleavage hydrodissection, endocapsular supporting devices, Osher's slow-motion phacoemulsification technique, tangential stripping motion, and use of foldable acrylic IOL [16, 17].

In a randomized controlled trial in India, MSICS has been shown to be as effective and safe as phacoemulsification with a small difference in uncorrected visual acuity and astigmatism [11]. It is faster, less expensive, and less technology dependent than phacoemulsification [18]. Moreover, it also provides closed stable chamber with a well-expanded and stable capsular bag for greater control, as well as minimizing further vitreous loss and therefore, risk of retinal detachment, glaucoma, and other complications [14].

Our study compared phacoemulsification and MSICS (Blumenthal technique) in subluxated cataracts in a randomized controlled case series. In 90.0% cases of phacoemulsification and 76.67% cases of SICS, the procedure was performed successfully without the need of major additional procedure and implantation of endocapsular supporting device and posterior chamber IOL although, this difference was not significant (*P* = 0.14).

Intraoperative complications in phacoemulsification were noted in 6 (20%) cases. Most common was inadvertent increase in zonular dehiscence (4 cases). In three cases, it occurred during chopping, out of which two required lensectomy with SFIOL and one case was managed with the insertion of two-point fixation Cionni ring. In one case, during implantation of single-point fixation CTR, zonular dehiscence increased during rotation into capsular bag. So the device was removed as it failed to provide stability and lensectomy with SFIOL was done. In another patient, capsulorhexis escaped, for which lensectomy with SFIOL was done. One patient had small capsulorhexis, which during postoperative follow up lead to development of capsular phimosis.

**Figure 1:**
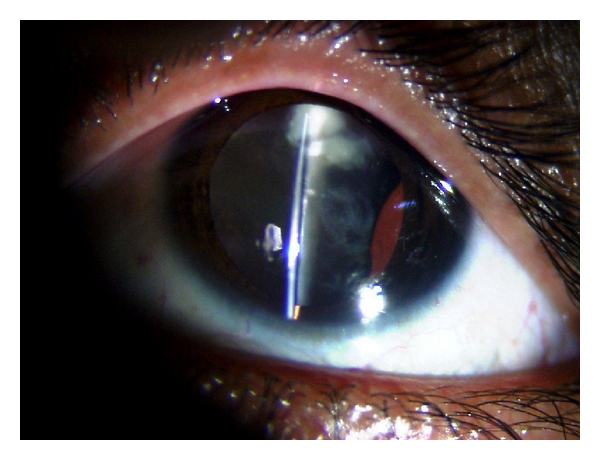
Lens coloboma with 180 degree subluxation.

 Intraoperative complications in MSICS were more frequent than phacoemulsification group and occurred in 11 (36.67%) cases, although this difference was insignificant (*P* = 0.15). The most frequent was increased zonular dehiscence during nuclear prolapse and escaped capsulorhexis with four cases each. In three cases where zonular dehiscence increased, nuclear sclerosis of grade 3 was present, and hence prolapsing a bigger nucleus out of bag was difficult which resulted in increase dehiscence. In one case, increase in zonulysis occurred in region of ACM, and this was managed with a placement of a two-point fixation Cionni ring. The other noted complication was escaped capsulorhexis, probably related to attempt at a larger capsulorhexis which is a prerequisite to prolapse the nucleus out of bag in MSICS. The capsular injury was noted in one case during the insertion of a single-point fixation Cionni ring. In two cases, nucleus failed to prolapse out of bag. In one such case, nucleus failed to rotate due to the presence of lens coloboma (Figure 1) and lensectomy was required. The inability to rotate nucleus was also encountered in fellow eye of the same patient during phacoemulsification but was overcome by dividing nucleus into pieces. This has been previously reported by Mizuno et al. [19], who recommend the use of endocapsular device in such a case. A second case, in which nuclear prolapse was impossible, had nuclear sclerosis of grade 4 and zonulysis of 190 degree. After the difficult insertion of a two-point fixation Cionni ring, the nucleus edge got stuck in the eyelet and had to be managed by intracapsular extraction and placement of SFIOL. Therefore, the implantation of the double point fixation device in cases with the grade-four nucleus could be difficult and may hinder the nuclear prolapse out of bag. The use of Ahmed capsular tension segment in such cases can provide effective lens stabilization before nucleus management [16].

The statistical analysis of intraoperative complications (Table 3) in relation to grade of nucleus in each group, showed that in group B (MSICS) they were significantly related to ≥3 grade of nucleus (*P* = 0.009), but the intergroup difference was insignificant.

We also compared the implantation of intended capsular device based on grade of nucleus and degree of zonulysis (Tables 4 and 5). Within each group, this was found to be significantly related to the grade of nucleus (*P* = 0.04  and  0.004 for groups A and B, resp.) and failure to implant an intended capsular device was seen more often in nucleus grade ≥3 in both the groups, although difference between the two groups was insignificant. The implantation of intended capsular device was not found significantly related to the degree of zonulysis, but in 3 out of 4 cases in SICS with zonulysis ≥180 degree, there was failure to implant an intended capsular device.

The most common long-term complication reported with the use of endocapsular supporting device is posterior capsule opacification (PCO) [20]. As follow up in our study was only 3 months, we did not compare the PCO rates in our series. We noted difficulty in performing the aspiration of cortical matter in cases where endocapsular supporting device was used. The visually significant postoperative complications noted in our series were cystoid macular edema (CME) and vitritis in each group. CME seen in MSICS group was probably caused by the increased manipulation of the iris as this case required the replacement of already placed single Cionni with double Cionni ring. Although CME in phacoemulsification group was not related to any cause. The visually insignificant complications were low-grade anterior uveitis (one case in each group), and transient vitreous haemorrhage was noted in two patients in MSICS group in which transscleral suture fixation of the posterior chamber IOL was done.

The limitation of our study is short-term followup. Few studies have reported the long-term results of endocapsular devices with rate of IOL dislocation ranging from 5.4 to 8.5% [21, 22]. With SFIOL, complications like suture rupture can occur in 6% of eyes at mean of 4.9 years [15] and up to 24% can have IOL dislocation after 7–10 years [23]. In a histologic study [24], IOL stability was the result of intact scleral sutures and not to fibrous encapsulation nor correct placement of the haptic in the ciliary sulcus. As a result, IOL dislocation is likely to occur if sutures are inadvertently removed or if suture fatigue occurs [25].

**Figure 2:**
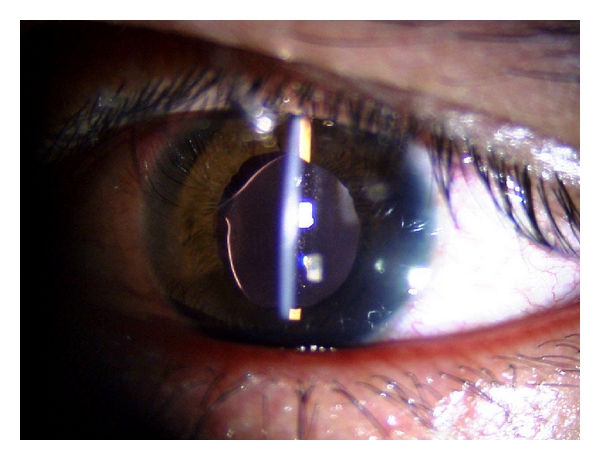
Transscleral suture fixated IOL decentration in group B.

IOL decentration was seen in one case in MSICS where SFIOL implantation was done due to escaped capsulorhexis (Figure 2). The capsular phimosis, probably caused by small size capsulorhexis and retained lens matter, was present in one case of phacoemulsification group, which led to the IOL tilt.

We did not relate astigmatism between the groups, because incision site varied in each patient according to the area of zonulysis. Postoperatively, both groups achieved good visual outcome, considering WHO definition of visual impairment as vision worse than 20/60 (equivalent to 0.33) [12], there were 28 patients (93.34%) in phacoemulsification and 27 patients (90%) in MSICS who were benefitted with the surgery.

## 5. Conclusions

Capsular bag retention in subluxated lenses is possible in 90% cases of phacoemulsification and 76.67% cases of MSICS. Both techniques achieved excellent visual outcome. The most common intraoperative complication noted in phacoemulsification was increased zonulysis and that in MSICS was increased zonulysis and escaped capsulorhexis. MSICS was performed with difficulty in cases of severe subluxation (>180), larger grade of nucleus (≥3), and lens coloboma. This was due to a larger-sized capsular opening required for prolapse the nucleus out of the bag. With greater zonulysis, there is more difficulty in creating a large capsular opening. During a follow-up period of 3 months, both the techniques were comparable in terms of BCVA, complications, and IOL decentration/tilt.

## Figures and Tables

**Table 1:** Preoperative comparison of two groups.

Characteristics	Group A	Group B	Chi square, *P* value
Patients (*n*)	30	30	
Mean age (yrs)^¤^	41.80 ± 12.80	39.86 ± 12.75	0.42, 0.51
Sex^†^			
Male	24 (80%)	20 (66.67%)	1.34, 0.24
Female	6 (20%)	10 (33.34%)	
Mean Preoperative BCVA (Decimal Acuity)^¤^	0.14 ± 0.10	0.13 ± 0.10	0.01, 0.91
Degree Of Subluxation^†^			
<90	8 (26.67%)	9 (30.0%)	0.08, 0.77
90–180	18 (60.0%)	17 (56.67%)	0.07, 0.79
>180^#^	4 (13.34%)	4 (13.34%)	—, 1.0
Cause^†^			
Trauma	17 (56.7%)	21 (70%)	1.13, 0.28
Idiopathic	11 (36.7%)	6 (20%)	2.02, 0.15
Other (congenital)^#^	2 (6.6%)	3 (10%)	—, 1.0
Grade of nucleus^¤^			
1	6 (20%)	7 (23.34%)	0.13, 0.98
2	13 (43.34%)	13 (43.34%)	
3	10 (33.34%)	9 (30.0%)	
4	1 (3.34%)	1 (3.34%)	
MEAN ± SD	2.2 ± 0.80	2.13 ± 0.81	0.10, 0.74

^¤^Mann Whitney test used (due to not normal distribution of variables) with Kruskal-Wallis *H* (equivalent to chi square).

^†^Mantel Haenszel test used.

^#^If expected cell value < 5, two-tailed *P* value using Fisher exact test was calculated.

**Table 2:** Postoperative comparison of two groups.

Characteristics	Group A	Group B	Chi square, *P* value
Devices for lens Stability^†^			
CTR/M-CTR	27 (90%)	23 (76.67%)	
None	3 (10%)	7 (23.33%)	1.89, 0.16
Successful implantation of intended device^†^			
Yes	25 (83.34%)	20 (66.67%)	2.19, 0.14
No	5 (16.67%)	10 (33.34%)	
Additional procedure^†^			
None	21 (70%)	17 (56.67%)	1.15, 0.28
Minor	6 (20%)	6 (20%)	0, 1.0
Major	3 (10%)	7 (23.34%)	1.89, 0.17
Intraoperative complications^†^	6 (20%)	11 (36.67%)	2.02, 0.15
Postoperative complications^†^	4 (13.34%)	5 (16.67%)	0.13, 0.72
Postoperative Corrected BCVA (decimal acuity)^¤^			
<0.3	2 (6.7%)	3 (10.0%)	0.26, 0.87
0.3–0.5	9 (30.0%)	8 (26.7%)	
>0.5	19 (63.3%)	19 (63.3%)	
MEAN ± SD	0.66 ± 0.23	0.68 ± 0.28	0.11, 0.73
IOL decentration (mm)^#^			
<1 mm	30 (100%)	29 (96.7%)	—, 1.00
>1 mm	0	1 (3.3%)	
IOL TILT (degree)^#^			
<15	29 (96.7%)	30 (100%)	—, 1.00
>15	1 (3.3%)	0	

^¤^Mann Whitney test used (due to not normal distribution of variables) with Kruskal-Wallis *H *(equivalent to chi square).

^†^Mantel Haenszel test used.

^#^If expected cell value <5, two-tailed *P* value using Fisher exact test was calculated.

**Table 3:** Distribution of intraoperative complications according to the grade of nucleus in two groups.

Grade of nucleus	Intended capsular device implantation				Statistical significance between two groups in relation to grade of nucleus
	Group A		Group B		
	Yes	No	Yes	No	
≤2	2	17	3	17	*P* = 1.00
≥3	4	7	8	2	*P* = 0.08

Statistical significance between nucleus grade within the group	*P* = 0.15		*P* = 0.0009		

**Table 4:** Intended capsular device implantation according to the grade of nucleus in two groups.

Grade of nucleus	Intended capsular device implantation				Statistical significance between two groups in relation to grade of nucleus
	Group A		Group B		
	Yes	No	Yes	No	
≤2	18	1	17	3	*P* = 0.6
≥3	7	4	3	7	*P* = 0.19

Statistical significance between nucleus grade within the group	*P* = 0.04		*P* = 0.004		

**Table 5:** Intended capsular device implantation according to the degree of zonulysis in two groups.

Degree of zonulysis	Intended capsular device implantation				Statistical significance between two groups in relation to degree of zonulysis
	Group A		Group B		
	Yes	No	Yes	No	
≤180	22	4	19	7	*P* = 0.31
≥180	3	1	1	3	*P* = 0.48

Statistical significance between degree of zonulysis within the group	*P* = 0.53		*P* = 0.09		
